# A high sensitivity Cherenkov detector for prompt gamma timing and time imaging

**DOI:** 10.1038/s41598-023-30712-x

**Published:** 2023-03-03

**Authors:** Maxime Jacquet, Saba Ansari, Marie-Laure Gallin-Martel, Adélie André, Yannick Boursier, Mathieu Dupont, Jilali Es-smimih, Laurent Gallin-Martel, Joël Hérault, Christophe Hoarau, Johan-Petter Hofverberg, Daniel Maneval, Christian Morel, Jean-François Muraz, Fabrice Salicis, Sara Marcatili

**Affiliations:** 1 Univ. Grenoble Alpes, CNRS, Grenoble INP, LPSC-IN2P3, 38000 Grenoble, France; 2 Aix-Marseille Univ, CNRS/IN2P3, CPPM, Marseille, France; 3 Ion beam application SA, 3, chemin du Cyclotron, 1348 Louvain-La-Neuve, Belgium; 4 Centre Antoine Lacassagne, 06200 Nice, France

**Keywords:** Imaging techniques, Biological physics

## Abstract

We recently proposed a new approach for the real-time monitoring of particle therapy treatments with the goal of achieving high sensitivities on the particle range measurement already at limited counting statistics. This method extends the Prompt Gamma (PG) timing technique to obtain the PG vertex distribution from the exclusive measurement of particle Time-Of-Flight (TOF). It was previously shown, through Monte Carlo simulation, that an original data reconstruction algorithm (Prompt Gamma Time Imaging) allows to combine the response of multiple detectors placed around the target. The sensitivity of this technique depends on both the system time resolution and the beam intensity. At reduced intensities (Single Proton Regime—SPR), a millimetric proton range sensitivity can be achieved, provided the overall PG plus proton TOF can be measured with a 235 ps (FWHM) time resolution. At nominal beam intensities, a sensitivity of a few mm can still be obtained by increasing the number of incident protons included in the monitoring procedure. In this work we focus on the experimental feasibility of PGTI in SPR through the development of a multi-channel, Cherenkov-based PG detector with a targeted time resolution of 235 ps (FWHM): the TOF Imaging ARrAy (TIARA). Since PG emission is a rare phenomenon, TIARA design is led by the concomitant optimisation of its detection efficiency and Signal to Noise Ratio (SNR). The PG module that we developed is composed of a small PbF$_{2}$ crystal coupled to a silicon photoMultiplier to provide the time stamp of the PG. This module is currently read in time coincidence with a diamond-based beam monitor placed upstream the target/patient to measure the proton time of arrival. TIARA will be eventually composed of 30 identical modules uniformly arranged around the target. The absence of a collimation system and the use of Cherenkov radiators are both crucial to increase the detection efficiency and the SNR, respectively. A first prototype of the TIARA block detector was tested with 63 MeV protons delivered from a cyclotron: a time resolution of 276 ps (FWHM) was obtained, resulting in a proton range sensitivity of 4 mm at 2$\sigma$ with the acquisition of only 600 PGs. A second prototype was also evaluated with 148 MeV protons delivered from a synchro-cyclotron obtaining a time resolution below 167 ps (FWHM) for the gamma detector. Moreover, using two identical PG modules, it was shown that a uniform sensitivity on the PG profiles would be achievable by combining the response of gamma detectors uniformly distributed around the target. This work provides the experimental proof-of-concept for the development of a high sensitivity detector that can be used to monitor particle therapy treatments and potentially act in real-time if the irradiation does not comply to treatment plan.

## Introduction

Protons have a very peculiar dose deposition profile compared to X-rays, with a sharp maximum at the end of their range (called Bragg peak), a limited entrance dose and nearly zero dose deposition after the Bragg peak^1^. These figures seem ideal to deliver highly conformal irradiations with a reduced number of fields and the highest selectivity. However, while the physical advantage of Proton Therapy (PT) is widely acknowledged, PT is still far from reaching its full potential. One of the most compelling open issues in PT is the reduction of irradiation uncertainties^2,3^. The main reason for this holdup comes from the objective technical complexity of predicting and verifying the proton path in the patient.

Some imaging devices have been proposed in the past to monitor the proton range *in vivo*, which are modified versions of classical nuclear medicine imaging modalities^4–10^, while other more original approaches do not provide the full PG profile (e.g. prompt gamma spectroscopy^11^, PG integral counts^12,13^). The existing methods have been extensively described in multiple reviews^3,14,15^. All these approaches exploit the spatial and/or temporal and/or energetic correlation of secondary particles emitted as a result of proton interactions with the biological tissue^3,16^. Among them, those based on the detection of Prompt Gamma (PG) rays resulting from non-elastic nuclear collisions in the patient are of particular interest to achieve a real-time measurement of the proton range and to allow stopping/correcting the treatment procedure from its very beginning in case a deviation from treatment planning is detected. Thanks to the prompt nature of these emissions, it is in principle possible to accomplish a statistically significant measurement of the proton range from the first irradiation spot. However, PG emission being a rare phenomenon (PG yield is $\lesssim$10$^{-2}$ PGs/proton/cm, with significant variations among models^17^), the development of high sensitivity PG detectors is crucial to obtain a real-time information. The required sensitivity can only be achieved by concurrently improving the intrinsic resolution of the measurement at a single event scale, and increasing the system detection efficiency to boost the measurement statistics.

With these goals in mind we have recently proposed a new modality to measure the PG vertex distribution *in vivo*: Prompt Gamma Time Imaging (PGTI)^18^. With PGTI, the overall Time-Of-Flight (TOF) of the proton ($T_{p}$) followed by the PG ($T_{PG}$) is first measured, as in the conventional PG Timing (PGT) approach^19,20^. Then, as both $T_{p}$ and $T_{PG}$ depend on the PG vertex coordinates $\textbf{r}_v$, the PGTI reconstruction algorithm retrieves the latter from the following equation:

1 $$\begin{aligned} TOF=T_{p}(\textbf{r}_v)+T_{PG}(\textbf{r}_v,\textbf{r}_d) \end{aligned}$$

where *TOF* is experimentally measured and $\textbf{r}_d$ are the PG hit coordinates at the detector level, or the detector position coordinates if the detector is small enough. Briefly, PGTI allows to convert the measured TOF distribution into a spatial distribution, by performing an event-by-event deconvolution of the PG TOF $T_{PG}(\textbf{r}_v,\textbf{r}_d)$. The direct consequence of this approach is to enable the readout of multiple detectors evenly distributed around the target: a capability that can be exploited to build a monitoring system that is sensitive to proton beam deviations both along the beam axis and in the transverse plane. The expected sensitivity of this technique has already been evaluated through Monte Carlo (MC)^18^ for different beam intensities.

A PGT-based detector naturally offers a high detection efficiency (of the order of $10^{-3}$) as no collimation system is needed, and it is therefore a good candidate for real-time monitoring. In order to push the system TOF resolution, and therefore its spatial resolution, we have proposed to use a fast, diamond-based beam monitor operated in single proton regime^21^ to tag in time each incident proton separately. Such a system, read-out in time coincidence with a conventional gamma detector, has already allowed us to reach TOF resolutions of 237 ps (FWHM) in a previous experiment with 68 MeV protons^22^.

Our current focus is to develop a fast gamma detector dedicated to PGTI^23^. The TIARA (TOF Imaging ARrAy) detector will be composed of approximately 30 independent modules evenly distributed around the target to achieve 4$\pi$ coverage. Each module will be composed of a $\sim$1 cm$^3$ monolithic Cherenkov radiator (PbF$_2$) read by Silicon PhotoMultipliers (SiPM). This system is designed to allow the measurement of the PG time of arrival with excellent time resolution, and its hit position with a spatial resolution limited to the Cherenkov crystal size. The use of a pure Cherenkov radiator offers several advantages compared to the use of more conventional scintillation detectors. The Cherenkov emission process is inherently faster than the scintillation, which favours temporal resolution. At the same time, Cherenkov radiators generally have higher effective Z than scintillators, which improves photon interaction probability. Nevertheless, we will show that their greatest advantage lies in their relatively insensitivity to neutrons, which allows to set a natural cut-off on one of the largest sources of background in proton-therapy monitoring, therefore increasing the Signal to Noise Ratio (SNR) of the measurement and reducing signal pile-up.

We have shown by MC simulation how this detector can be operated in three different regimes depending on the beam intensity^18^. In Single Proton Regime (SPR), the beam intensity is reduced during the first irradiation spot to approximately 1 proton per bunch. Under this regime, the statistics available for the proton range measurement is limited, but the excellent time resolution achievable (of the order of 235 ps FWHM) ensures a measurement sensitivity of the order of 1 mm at 2$\sigma$ for 10$^{8}$ protons of 100 MeV and a simulated detection efficiency of 0.6%. Alternatively, a sensitivity of 3 mm at 2$\sigma$ was also predicted for 10$^{7}$ incident protons.

At nominal beam intensity (from $\sim$2000 to $\sim$2 million protons every 16 ns at isocenter^37^ for the clinical accelerator used in this work), the time resolution is ultimately limited by the bunch time-width of the proton beam as it is impossible, with the current beam monitor, to establish which proton in the bunch has generated the detected PG. Nevertheless, the loss of time resolution is substantially compensated by the increased measurement statistics, and sensitivities of a few mm can still be reached, depending on the number of PGs included in the monitoring procedure: in our previous, work we estimated a proton range sensitivity of 2 mm (at 2$\sigma$) for 10$^{9}$ incident protons^18^.

Finally, at very high intensities, a one-value measurement of the proton beam displacement can still be obtained by computing the centre of gravity of the TIARA detection modules. With this approach, no assumption should be made on the detector time resolution as only the counts registered in each module (and their absolute position) are needed in the reconstruction formula. A sensitivity of 2 mm (at 2$\sigma$) on the proton beam lateral displacement was estimated for 10$^{8}$ incident protons, whereas the method is less sensitive to distal proton range shifts.

The present work completes our previous simulation studies by confirming the hypotheses made and demonstrating the experimental feasibility of PGTI in SPR. In this case, the reduced intensity regime allows to characterise the inherent performances of the detection module, that would otherwise be affected by the time properties of the accelerator. Through the results obtained in two experiments carried out with 63 and 148 MeV protons, the performances of the detector in terms of time resolution will be presented in the first section. We will also show that the gamma-ray energy measurement can be disregarded when using extremely fast detectors, and how a millimetric sensitivity on proton range measurement could be achieved with an unprecedentedly low PG statistics. In the second section, we will show the advantages of performing PGTI with detectors placed at multiple angular positions with the aim to reach a uniform sensitivity in the whole field of view. Finally, the different sources of background affecting our Cherenkov-based detector will be discussed in the third section.

## Results

### Detector characterisation with 63 MeV protons

A detector module based on a 1$\times$1$\times$1 cm$^{3}$ PbF$_2$ Cherenkov radiator coupled to a 3$\times$3 mm$^{2}$ MPPC from Hamamatsu (S13360-3050CS) read by a commercial preamplifier from Advansid [https://advansid.com/products/product-detail/asd-ep-eb-pz] was conceived at LPSC (Laboratoire de Physique Subatomique et Cosmologie). The module was tested at the CAL (Centre Antoine Lacassagne) MEDICYC facility^24^ with protons of 63 MeV delivered in bunches of 4 ns duration every 40 ns period.

**Figure 1:**
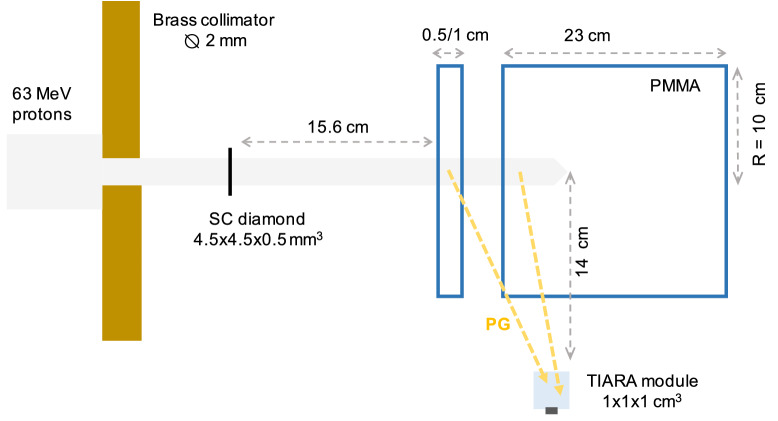
Set-up of the experiment carried out at the MEDICYC facility with 63 MeV protons. A first version of the TIARA module, composed of a 1 cm$^3$ PbF$_2$ crystal coupled to a 3$\times$3 mm$^2$ SiPM and facing the Bragg peak region at 90$^{\circ }$, was tested in time coincidence with a sc diamond detector of $4.5\times 4.5\times 0.55$ mm$^3$ volume. For the time and energy response characterisation, only PG signals from the thin PMMA target (cylinder of 10 cm radius) were considered: repeated measurement were carried out with either a 5 mm or a 1 cm thick target and the results were then averaged. The second target (cylinder of 10 cm radius and 23 cm thickness) was employed in the measurement of the proton range sensitivity; initially placed at 3 cm distance from the thin target, it was progressively moved by 2, 4, 6 and 10 mm in order to induce an artificial shift in the proton range. All the targets have a density of 1.19 g/cm$^3$.

A first cylindrical PMMA target (density = 1.19 g/cm$^3$) of 0.5 cm (or 1 cm) thickness and 10 cm radius (cf. Fig. 1) was employed to characterise the module energy and time response, followed by a cylindrical PMMA block of 23 cm thickness (enough to stop the beam) and 10 cm radius, placed at 3 cm distance from the thinner PMMA target. A single crystal (sc) diamond detector of $4.5\times 4.5\times 0.55$ mm$^3$ volume from Element6 [https://e6cvd.com/] was used as a beam monitor to tag in time the incident protons. The diamond was read-out on both faces using a commercial C2 preamplifier from Cividec [https://cividec.at/electronics-C2.html]; the two signals were summed up for analysis in order to increase the SNR and therefore improve the time resolution as proposed by Curtoni et al.^25^. A 8 mm thick, 2 mm diameter brass collimator was used to match the beam size to the limited diamond detection surface. The effective (after collimation) beam intensity reaching the beam monitor was estimated *a posteriori* to amout $\sim$0.025 p/bunch from Poisson statistic considerations (see the Methods section). A time resolution of 156 ps (FWHM) was previously measured for sc-diamonds in the same energy range^22^ and similar experimental conditions. The gamma detector module was positioned on the side of the large PMMA target at 14 cm from the beam axis and facing the Bragg-peak region in the thick target (orthogonal to the beam). All signals were acquired in time coincidence with the beam monitor and then digitally sampled using a HDO6104A-MS LeCroy oscilloscope with 1 GHz bandwidth, 10 Gs/s and a 12 bit ADC. The analysis was performed offline. In order to obtain a perfect SPR, the small fraction of 2-protons events acquired was cut-off during the analysis.

#### Energy response

**Figure 2:**
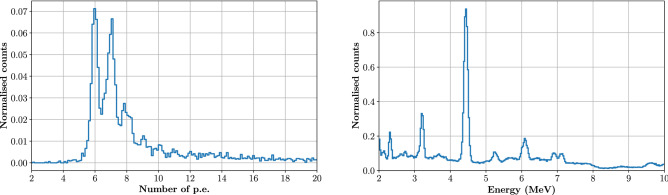
The left plot shows the histogram of the energy deposited in the TIARA module (1 cm$^3$ PbF$_2$ crystal coupled to a 3$\times$3 mm$^2$ SiPM and placed at 90$^{\circ }$ respect to the beam) expressed as the integral of the SiPM signal. PGs are generated by 63 MeV protons impinging on the two PMMA targets described in Fig. 1. The first peak in the histogram corresponds to 6 p.e; the 7, 8, 9 p.e. peaks are also visible: a median number of 7 p.e. per PG were detected. The right plot shows the expected energy spectrum of PGs obtained by MC simulation for comparison. The simulation is performed with the Geant4.10.4.p02 toolkit implementing the QGSP-BIC-EMY physics list and reproducing the PMMA target and beam parameters used in the experiment.

Signals from the TIARA module were integrated to record the detector energy response as shown in Fig. 2, left. On the right, the simulated energy distribution of PGs from 63 MeV protons impinging on a PMMA target is shown for comparison. The two spectra are clearly unrelated as the TIARA module does not allow to establish the PG incident energy. As a result, none of the intense, characteristic PG emission lines visible in the right plot can be distinguished in the left plot. Actually, because of the limited detection volume, the gamma ray energy is not fully deposited in the detector. As a consequence, multiple interactions of mono-energetic gamma rays may result in a wide range of deposited energies. This phenomenon, combined to the relatively low light yield of Cherenkov radiators in the 2−10 MeV range, results in the typical SiPM single photo–electron (p.e.) spectrum shown in Fig. 2 (left), in which single Cherenkov photons are literally counted by the device.

An acquisition threshold of 6 p.e. was applied to the gamma detector signal in order to reduce the probability of triggering on SiPM dark counts. For the same purpose, the coincidence with the beam monitor was also imposed for data acquisition. A median number (*N*) of 7 p.e. was detected, corresponding to the amount of Cherenkov photons detected by the SiPM. The value of *N* indirectly affects the module time resolution as the SiPM contribution roughly goes as $SPTR/\sqrt{N}$^26^, where *SPTR* is the intrinsic Single Photon Time Resolution (SPTR) of the SiPM.

**Figure 3:**
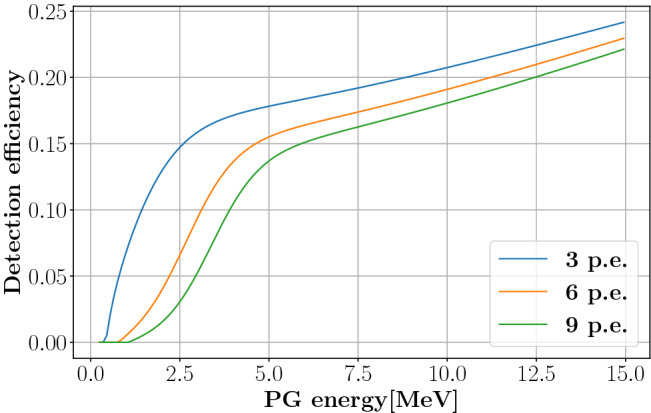
Intrinsic detection efficiency (neglecting geometrical efficiency) of a PG detection module composed of a 1 cm$^3$ PbF$_2$ crystal coupled to a 3$\times$3 mm$^2$ SiPM. The three functions were obtained from MC simulation using the Geant4.10.4.p02 toolkit with the QGSP-BIC-EMY physics list to establish the PG interaction probability, while the UNIFIED model was applied to model the interactions of Cherenkov photons inside the crystal. Three different thresholds of 3, 6 and 9 p.e. were considered.

The poor proportionality between incident and deposited PG energy mainly affects the determination of the acquisition threshold as, at low energies, the module detection efficiency dramatically depends on the PG incident energy. This can be estimated through MC simulation (see the Methods section). Neglecting the geometrical contribution, the intrinsic detection efficiency of a PG module as the one described in Fig. 1 depends on two contributions: the probability for the PG to interact in the crystal and the probability that this interaction produces more than $N_{th}$ photoelectrons, with $N_{th}$ being the threshold in p.e. applied for data acquisition. Figure 3 shows the detection efficiencies as a function of PG energy obtained by MC simulation for three different thresholds of 3, 6 and 9 p.e. It can be observed that the threshold does not provide a sharp energy cut-off. For example, using a threshold of 6 p.e. gives a 5% probability of detecting PGs with an energy of 2 MeV.

We will show in the next paragraphs that the lack of energy resolution does not compromise the proton range measurement sensitivity when a very high time resolution is achieved as long as the detection efficiency is properly taken into account.

#### Coincidence time resolution

**Figure 4:**
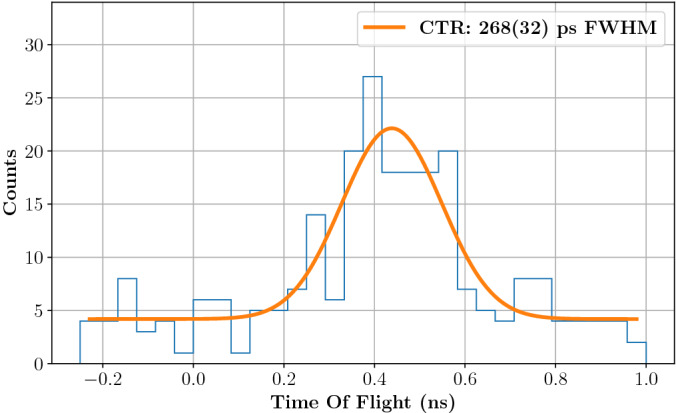
TOF distribution obtained with the 5 mm thick, 10 cm radius PMMA target irradiated by 63 MeV protons. The PG detector consisted of a 1 cm$^3$ PbF$_2$ crystal coupled to a 3$\times$3 mm$^2$ SiPM and placed at 90$^{\circ }$ respect to the beam. Data are fitted with a gaussian distribution convolved with a uniform distribution of 51 ps width. The resulting FWHM of 268 ps (114 ps rms) corresponds to the gaussian distribution FWHM and it can be interpreted as the system CTR.

The time stamps of both the diamond detector and the TIARA detection module have been recorded to obtain the proton plus gamma *TOF* variable in Eq. (1). The two diamond signals (one from each face) are first summed-up to increase their SNR, then a digital Constant Fraction Discrimination (CFD) with a 50% CF value is applied to both the PG and the diamond detectors. The resulting PG TOF distribution is presented in Fig. 4 for events corresponding to single protons in the beam monitor.

This distribution is a measure of the system Coincidence Time Resolution (CTR): more precisely, its width results from the convolution of the system intrinsic time resolution and the proton transit time in the 5 mm PMMA target. The latter can be approximated by a uniform distribution of $\sim$51 ps width (from Geant4 simulations). After deconvolution of the proton transit time, and the suppression of the flat background, the best system time resolution is estimated to be 268 ± 32 ps (FWHM) for data in Fig. 4. The same analysis was carried out independently on three TOF distributions acquired with either the 5 mm or the 1 cm thick targets; the averaged time resolution obtained from these repeated experiences is of 315 ± 40 ps (FWHM). The CTR can also be interpreted as the quadratic sum of the beam monitor’s and the PG detector’s time resolutions under the hypothesis that the two contributions are independent and gaussians. Since the diamond time resolution was measured to be 156 ps (FWHM) in similar conditions^22^, the TIARA detection module time resolution can be estimated to approximately amount 276 ps (FWHM). Therefore, for this detection module design and for 63 MeV protons, the system CTR is dominated by the PG time measurement.

#### Study of PGT sensitivity

**Figure 5:**
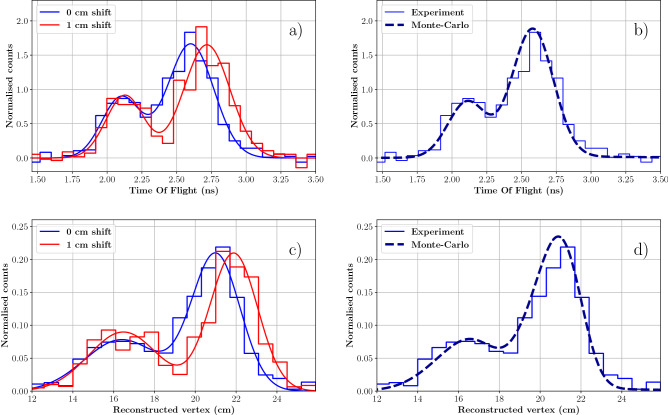
PGT profiles (top) and PGTI reconstructed profiles (bottom) obtained from the PMMA targets described in Fig. 1 irradiated with 63 MeV protons. In (**a**) the experimental TOF profile obtained for the reference geometry (in blue) is compared to the one obtained after engendering a proton range shift of 1 cm (in red). The two histograms are fitted with a double gaussian fuction to improve readability; the fit was not used for analysis. In (**b**), the simulated reference profile (dashed line) is compared to the corresponding experimental data. In (**c**) the two profiles shown in (**a**) are reconstructed with the PGTI algorithm to convert them into the space domain. In (**d**) the reconstructed simulated (dashed line) and experimental (continuous line) reference profiles, corresponding to data in figure (**b**) are presented. The experimental profiles are obtained with a PG module composed of a 1 cm$^3$ PbF$_2$ crystal coupled to a 3$\times$3 mm$^2$ SiPM and placed at 90$^{\circ }$ respect to the beam. For the simulation of the reference profile, we used the Geant4.10.4.p02 toolkit with the QGSP-BIC-EMY physics list and the UNIFIED model.

The 1 cm thick PMMA target was placed downstream the diamond detector (cf. Fig. 1) with the thick target positioned at 3 cm distance. This configuration (considered as the reference geometry) was employed to simulate an air cavity heterogeneity in a uniform anatomy. The distance between the two targets was then progressively increased by 2, 4, 6 and 10 mm to reproduce an unpredicted variation in the anatomy and consequently a shift in the proton range. For each shift (0, 2, 4, 6 and 10 mm), the TOF distribution between the proton beam monitor and the PG detector was recorded. Figure 5a shows, as an example, the TOF histograms measured at 3 cm (0 mm shift) and 4 cm (10 mm shift) target-to-target distance. The two distributions are clearly separated: the distal fall-off of the 4 cm shift distribution is displaced towards higher TOFs according to the air cavity thickness introduced between the targets. The flat background contribution, due to the random coincidences between SiPM dark counts and protons interacting in the diamond, is subtracted before analysis, and only 1-proton signals in the beam monitor are considered. After background rejection, each of the 5 TOF distributions includes around 600 PG events. For this experiment, it is not possible to establish the number of protons delivered as the scope dead time when writing the waveforms’ values on disk considerably affects the total acquisition time. Nevertheless, previous MC simulations^18^ allow to estimate that 600 PG events would correspond approximately to 2 $\times$10$^{6}$ incident protons for the full 30-channels TIARA prototype.

The dashed curve in Fig. 5b represents the simulated PG profile in the reference geometry (0 mm shift), that was obtained taking into account the energy dependency of the PG module efficiency presented in Fig. 3 (see the Methods section). This curve is used as a term of comparison to perform relative measurements of the proton range shift according to the approach previously described^18,22^ and summarised in the methods section. This methodology measures the proton range shift only exploiting the distal region of the PG profiles without making any assumption on the profiles’ shape. The double gaussian fit (continuous lines in Fig. 5, left) are only presented as eye-guide but were not used for the analysis. In the right plot, an excellent agreement between the experimental and the simulated reference profiles can be observed, serving as a validation of the detector model developed in Geant4.

**Figure 6:**
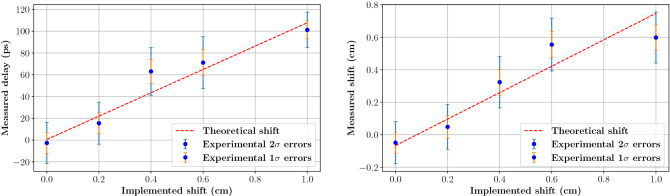
Range shift sensitivities obtained with a PMMA target including an air cavity ranging from 3 to 4 cm, and irradiated with 63 MeV protons. On the left, the proton range shift measured with the PGT technique in unit of time is compared to the actual shift implemented in the phantom. On the right, the PGTI reconstruction allows measuring the proton range shift directly in the space domain. The dashed red line corresponds to the theoretical correlation between the implemented shift and the measured parameter as obtained from MC simulation using the Geant4.10.4.p02 toolkit with the QGSP-BIC-EMY physics list and the UNIFIED model. Error bars represent the 1$\sigma$ (orange) and 2$\sigma$ (blue) statistical errors obtained by the bootstrap technique (see the Methods section). With both techniques, the TIARA detection module (1 cm$^3$ PbF$_2$ crystal coupled to a 3$\times$3 mm$^2$ SiPM and placed at 90$^{\circ }$ from the beam direction) permits to achieve a proton range shift sensitivity of 4 mm at 2$\sigma$ with a statistics of approximately 600 PGs.

The measured shifts are reported in Fig. 6, left, as blue experimental data points, as a function of the implemented target shift. The dashed red line represents the correlation between the measured time delay and the shift implemented in the geometry as obtained by MC simulation. Its slope amounts to 107 ps/cm, roughly corresponding to the proton speed at the exit of the thin target (the simulated value amounts to 109 ps/cm). The orange and blue error bars respectively summarise the 1$\sigma$ and 2$\sigma$ experimental errors obtained by bootstrapping methods (see methods section). The experimental errors on these data indicate that the TIARA detection module is able to measure a 4 mm proton range shift at 2$\sigma$ confidence level, from the exclusive measurement of TOF with a very low statistics of acquired PGs. The same data also show that a proton range sensitivity of 2 mm would be achievable at 1$\sigma$ confidence level.

#### Study of PGTI sensitivity

The five TOF datasets acquired for the different target shifts are reconstructed on an event-by-event basis according to Eq. (1) and following the methodology described in^18^. With this procedure, the TOF distributions in Fig. 5a are converted into PG profiles in the space domain providing straightforward means to directly measure the range shift in mm instead of ps (see Fig. 5c). In analogy to Fig. 5a, data are shown for the 3 and 4 cm air cavities. The time-to-space conversion is necessary to combine the response of multiple modules placed at different angular coordinates. Actually, the PG TOF depends on the relative position between the PG vertex and the PG detector and it must be deconvolved before summing-up TOF distributions obtained with different modules. In this work, only one TIARA detection module was available: our goal therefore was to demonstrate that PGTI can provide the same sensitivity than PGT.

The proton range shift was evaluated with respect to the simulated PGTI profile in reference conditions (dashed line in Fig. 5d) applying the same approach used for PGT distributions after background subtraction (see methods section). In analogy with PGT analysis, the measured proton range shift is reported in Fig. 6, right, against the applied cavity shift. The 1$\sigma$ and 2$\sigma$ statistical errors are also shown as orange and blue error bars, respectively. The same proton range sensitivity is observed for PGTI and PGT: a 4 mm range shift could be distinguished at 2$\sigma$ confidence level. Nevertheless, an offset of 0.66 mm is present on the PGTI dataset, as highlighted by the dashed red line. This curve represents the expected correlation between the measured and the implemented range shift as obtained by MC simulations; here its intercept has been adjusted to the data points to properly estimate the range offset. This offset results from the propagation, during reconstruction, of the systematic errors made when measuring the coordinates of the TIARA detection module and those of the beam monitor. In the future, this error can be easily minimised by imaging the target (or the patient) and the experimental set-up with a CT scanner in order to establish the detectors’ positions with sub-millimetric precision during the treatment planning phase. Despite this offset, the behaviour of the experimental points in Fig. 6, right, is still linear with a slope of 0.81, a value inferior to one as the current reconstruction is biased by the $T_{p}$ term (cf. Eq. 1) determined in reference conditions. This effect could be avoided with an iterative reconstruction approach, as the one proposed by Pennazio et al.^36^, that could establish offline the actual range shift. Nevertheless, in this work we focussed on an event-by-event reconstruction that can be implemented online during data acquisition, hence allowing to promptly stop the treatment if a significant shift is detected. To achieve this goal, we do not need the actual value of the proton range shift, but only to establish whether a statistically significant discrepancy with respect to the treatment plan exists or not.

### Detector characterisation with 148 MeV protons

A second version of the detector module was realised to improve the detection efficiency without compromising the time resolution. It is composed of a 1$\times$1$\times$2 cm$^3$ PbF$_2$ crystal coupled to a 6$\times$6 mm$^2$ MPPC from Hamamatsu (S13360-6075CS) and read by a custom preamplifier based on the design of Cates et al.^27^. The module was tested with 148 MeV protons at the ProteusOne S2C2 synchrocyclotron at CAL. S2C2 delivers protons in micro-bunches of 16 ns period with a 50% duty cycle^28^: this micro-structure is embedded in a macrostructure characterised by 8 $\mu$s pulses every ms. In this paper, the 8 $\mu$s beam structure will be referred to as proton pulse, whereas the 16 ns micro-structure will be mentioned as proton bunch. The beam profile is Gaussian with a measured standard deviation of 4.3 mm at 148 MeV^29^. The same sc-diamond detector used in the 63 MeV experiment, read on both sides using Cividec C2 preamplifiers, provided the time stamps for the incident protons, The effective intensity at the beam monitor level was arbitrarily set to a low value that was estimated *a posteriori* to amount $\sim$0.78 p/bunch on average.

**Figure 7:**
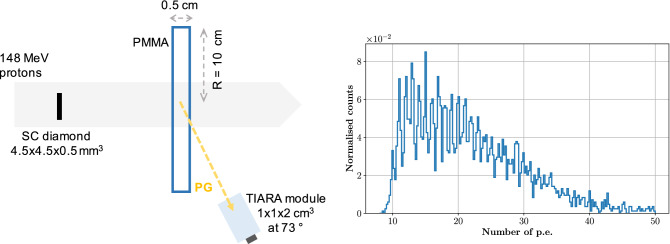
Left: experimental set-up for the CTR measurement carried out at the S2C2. A 5 mm thick, 10 cm radius PMMA target was used as a source of PGs to measure the CTR. Given the limited transit time of protons within the target, the PG source can be considered point-like. The beam size (4.3 mm $\sigma$) was larger than the diamond surface (4.5$\times$4.5 mm$^2$) as no collimator was employed. The TIARA detection module was composed of a 2 cm$^3$ PbF$_2$ crystal coupled to a 6$\times$6 mm$^2$ SiPM and placed at approximately 73$^{\circ }$ from the beam direction. The effective beam intensity at the beam monitor level was 0.78 p/bunch. Right: energy deposited in the TIARA detection module expressed as the integral of the SiPM signal. A threshold of 10 p.e. was applied for data acquisition, resulting in a median number of 21 p.e. detected.

In a first experiment (cf. Fig. 7, left), a 5 mm thick, 10 cm radius cylindrical PMMA target (density = 1.19 g/cm$^3$) was employed in a configuration similar to the one used at the MEDICYC accelerator in order to measure the system CTR. The TIARA module was placed close to the target axis in order to increase the geometrical efficiency, at approximately 73$^{\circ }$. Signals from all detectors were digitally sampled using a Wavecatcher module^30^ with 500 MHz bandwidth and a sampling rate of 3.2 Gs/s. The acquisition was triggered by the coincidence of the two detectors within a 15 ns time window. The analysis was performed offline. The SPR was ensured by selecting only 1-proton events in the diamond detector. Scattered protons directly detected by the SiPM in the PG module were rejected by pulse-shape analysis as they produce longer signals than those associated to Cherenkov events in the crystal.

**Figure 8:**
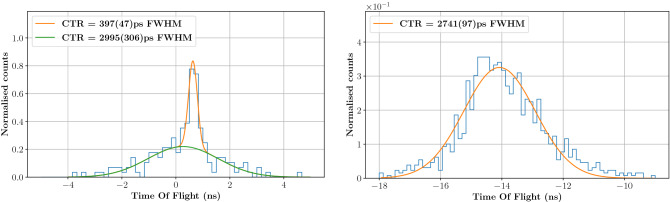
On the left, TOF distribution obtained from the irradiation of the 5 mm target. The gaussian peak with 397 ps (FWHM) corresponds to PGs generated by protons traversing the beam monitor; the large background signal corresponds to PGs generated by protons passing by the diamond detector. On the right, the TOF distribution obtained from random coincidences between the diamond and the PG detectors confirms the origin of the background on the left plot: it is a measure of the bunch-induced time resolution.

The energy response of the module is shown in Fig. 7, right: a median number of 21 p.e. is detected per each PG event when applying an acquisition threshold of 100 mV ($\sim$10 p.e.). This improvement, with respect to the median value of 7 p.e. obtained in the previous experiment (Fig. 2, left) is mainly due to the increased size of the SiPM that allows collecting a larger number of Cherenkov photons per PG event. The difference between the PG detector time stamp (obtained with a 5% CF threshold) and the diamond time stamp (obtained with a 50% CF threshold) was calculated to build the TOF distribution for the 5 mm target irradiation shown in Fig. 8. This distribution presents two clear components: (i) a well resolved gaussian peak (highlighted by the orange fit) that corresponds to PG events generated by protons crossing the diamond detector, and (ii) a broad background (highlighted by the green fit) associated to PG events from protons passing by the beam monitor.

The first peak directly provides a measurement of the system CTR with a value of 397 ps (FWHM). This CTR can also be interpreted as the quadratic sum of the diamond and the PG detector time resolutions. In a previous experiment, the intrinsic time resolution of the diamond was measured to be 360 ps (FWHM) when reading-out a single face of the detector. Under these conditions, the diamond time resolution is expected to be overestimated by 50–70 ps (FWHM) with respect to experiments in which signals from both sides are summed-up for analysis^25^. Despite the measured time resolution can not be directly compared to the CTR measured in this work, it still appears obvious that, for 148 MeV, the system CTR is now dominated by the beam monitor, with the TIARA detection module contributing for less than 167 ps (FWHM). This effect is expected, as an increase in the proton energy corresponds to a decrease in the energy deposited in the diamond: $\sim$0.9 MeV is deposited in a 0.55 mm thick diamond by 148 MeV protons instead of $\sim$1.7 MeV for 63 MeV protons.

The background component in the TOF distribution of Fig. 8 is a direct effect of the limited size of the beam monitor. Since no collimator was used in this experiment, only a fraction of the beam (approximately 20%) traverses the diamond detector. This means that, while the effective beam intensity was estimated to 0.78 p/bunch at the beam monitor level, the actual beam intensity is of the order of 4.7 p/bunch. Therefore the acquisition of part of the events may be triggered by the coincidence of a PG (or another secondary particle) with a random proton that has not produced the gamma ray (or the secondary particle). In other words, the background distribution represents the random coincidences between the PG detector and the beam monitor: its shape is not flat, because the beam time-structure is periodic with a nominal bunch width of 8 ns and a nominal standard deviation of $8/\sqrt{12}$ ns. The background could be fitted with a Gaussian distribution of 1.27 ± 0.13 ns sigma, a value that is consistent with the S2C2 micro-bunch standard deviation. This hypothesis is confirmed by a separate, more direct measurement of the bunch width. A random coincidences TOF distribution was built between events from the PG module and the beam monitor triggering on the bunch arriving immediately before the 15 ns coincidence window. The distribution obtained, shown in Fig. 8, right, is compatible with the background in Fig. 8, left, with its mean value shifted by the micro-bunch period (16 ns). The standard deviation of this distribution is 1.17 ± 0.04 ns, in agreement with the measurement in Fig. 8 left.

**Figure 9:**
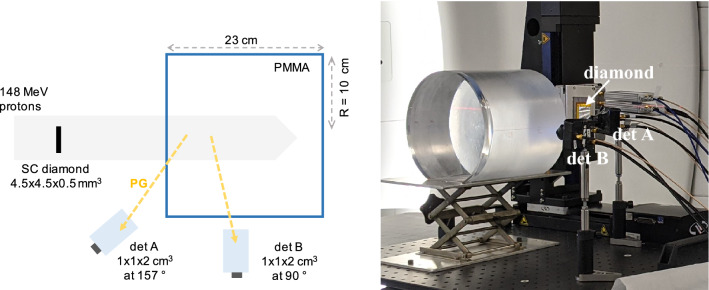
Experimental set-up for the PGT profile measurement at the S2C2 facility. The 23 cm thick, 10 cm radius PMMA target fully stopped the 148 MeV protons after a range of 13.4 cm. Two gamma detector modules were placed upstream the target (det A, at 157$^{\circ }$) and at the Bragg peak (det B, at 90$^{\circ }$). Each module was composed of a 2 cm$^3$ PbF$_2$ crystal coupled to a 6$\times$6 mm$^2$ SiPM. The effective beam intensity at the beam monitor level was 0.78 p/bunch.

**Figure 10:**
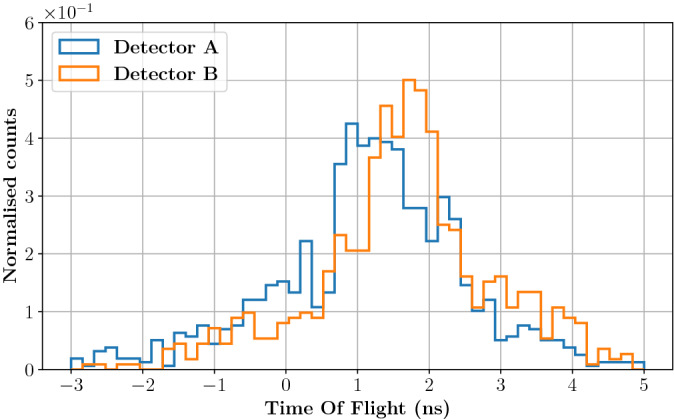
TOF distributions obtained with the thick PMMA target and with detector A (in blue) placed at 157$^{\circ }$ and detector B (in red) placed at 90$^{\circ }$ from the beam direction. The relevant signal is in the region between 0.5 and 3 ns. Outside this region, the background is mainly due to random coincidences caused by the limited size of the beam monitor. The two bumps located at (−1.0 $\div$ 0.5) ns and (2.9 $\div$ 4.1) ns for detectors A and B distributions, respectively, are associated to protons scattered in the beam detector.

In a second experiment, the thin target was replaced by a 23 cm thick, 10 cm radius PMMA target to stop 148 MeV protons after a 13.4 cm range. Two identical gamma detectors were placed close to the beam entrance (position A, at 157$^{\circ }$), and close to the Bragg peak (position B, at 90$^{\circ }$) both aiming at the Bragg peak region as shown in Fig.  9. During analysis, only 1-proton events were considered and scattered protons directly detected by the SiPM in the PG module were rejected by pulse-shape analysis. The TOF distributions measured for detectors A and B are presented in Fig. 10. The same effect described in Fig. 8 is visible here: because of the beam monitor limited size, a background associated to random coincidences is recognisable in both distributions. A second background component is present in the TOF ranges (−1.0 $\div$ 0.5) ns and (2.9 $\div$ 4.1) ns for detectors A and B distribution, respectively. These events are associated to scattered protons generating PGs in the gamma detector (most probably in the packaging) and will be discussed in detail in the next section. Nonetheless, the PG signal is still clearly detected between 0.5 and 3 ns for both detectors. Detector A is directed at the beam entrance while detector B is closer to the Bragg peak region. Thus, detector A has a larger solid angle for the measurement of PGs at the target entrance with a reduced efficiency for the Bragg peak region, whereas the opposite is true for detector B, explaining the different positions of the PG profiles maxima and their shapes. Traditionally, PGT detectors are placed at beam entrance to take advantage of the overall longer TOF, thus increasing the TOF measurement sensitivity^19,31^, and to avoid scattered protons that are mostly forward-directed. However, this approach limits the PG statistics acquired in the fall-off region where the proton range measurement is performed^20^ and it ultimately reduces the technique sensitivity; while the profile fall-off is very sharp for detector B, the one obtained with detector A is not well defined. Combining the readings of multiple detectors placed at different angular positions around the target is therefore the only way to achieve a uniform statistical efficiency and a uniform sensitivity throughout the whole proton range. This possibility could lead to further exploit the measured PG profile in order to assess anatomy variations in patients^32^, but it requires a dedicated reconstruction as the one proposed by PGTI in order to appropriately sum-up the response of multiple detectors. As a tangible example, TOF distributions acquired with detectors in position A and B are non-uniformly shifted and stretched in the time domain, according to the variation of the PG TOF all over the proton range. While the proton transit time in the target is, by definition, the same for the two detectors, the PG TOF varies from 165 to 507 ps for detector A (342 ps of relative PG delay), and from 329 to 407 ps for detector B (78 ps of relative PG delay) for PGs generated at the target entrance and for those generated at the Bragg peak respectively: the two TOF distributions cannot be summed-up in the time domain without causing blurring and a loss of resolution/sensitivity. Alas, in this experiment, the presence of the broad background from random coincidences in the beam monitor prevented PGTI data reconstruction. In fact, the current PGTI algorithm needs the system time resolution as an input parameter for the reconstruction^18^, and in presence of a double component it is impossible, for a single event, to establish the associated time resolution. Our current effort is in the development of a larger area beam monitor in order to cover the whole beam surface^33^.

### Background events in Cherenkov detectors

One of the hypotheses that has motivated the use of Cherenkov radiators for PG imaging is their insensitivity to neutrons. A PG detector is subjected to three main sources of background noise: neutron and neutron-induced gamma-rays that are time-correlated to the proton beam (i.e. originating in the nozzle or in the patient); neutron and neutron-induced gamma-rays from the environment; and protons scattered in the patient/target and, in our case, the beam monitor.

**Figure 11:**
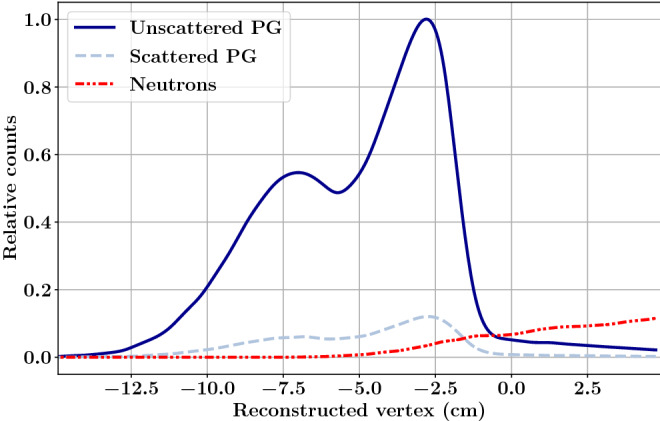
Vertex distributions of different secondary particles generated by a 100 MeV proton beam impinging on the spherical phantom head described in Jacquet et al.^18^. Data are obtained by MC simulation (Geant4.10.4.p02 toolkit with the QGSP-BIC-EMY physics list); the detector is not simulated. The contribution of PG scattered in the phantom is reported separately. It can be observed that their profile has the same shape as the one from unscattered PGs and therefore they constitute a valuable signal. The contribution of secondary neutrons (in red) is not constant and cannot be easily rejected by TOF without compromising the measurement of the PG profile fall-off. Data are taken from Jacquet et al.^18^.

From MC simulation^18^, it has already been demonstrated that, for most of the particles falling in the first category, the contribution is not flat, but it increases at the fall-off of the PG profile (see neutron contribution in Fig. 11, data from^18^). Thus, even if this component may be rejected by TOF selection^22^, still, the fall-off of the PG profile will be biased, with a direct consequence on the accuracy of the proton range measurement.

Conversely, non time-correlated background particles result by definition in a constant baseline in the TOF distribution. The absence of a collimator is pivotal in keeping the level of this environment noise negligible. The exploitation of a threshold process such as Cherenkov emission offers additional means for neutron rejection. Secondary particles produced by neutron scattering are too massive to reach the critical speed for Cherenkov emission; the same is true for scattered protons. Neutron capture, instead, requires neutron thermalisation; even if the neutron was detected through this process, its slow speed would guarantee an effective TOF selection. Basically, the only possible source of background in presence of a TOF-Cherenkov radiator comes from neutron-induced gamma rays that are not time-correlated or moderately correlated to the proton beam.

The locally constant background measured in the test with 63 MeV protons (Fig. 5a) provides an experimental evidence that the noise is not time-correlated in the region of interest for PG monitoring. Its frequency ($f_{noise}$) was estimated as the integral counts in the constant background divided by the acquisition duration, resulting in $f_{noise}$ = 1.46 ± 0.02 Hz. Under the assumption that the noise is mostly due to the SiPM dark count rate ($DCR_{SiPM}$), $f_{noise}$ should be equal to the rate of random coincidences $DCR_{coinc}$ between proton triggers in the diamond ($f_{dia}$) and $DCR_{SiPM}$. $DCR_{coinc}$ can be estimated as:

2 $$\begin{aligned} DCR_{coinc}=f_{dia} \times DCR_{SiPM} \times 2\tau \end{aligned}$$

where $2\tau$ is the coincidence window width of 20 ns. From the proton beam intensity of 0.025 ± 0.002 p/bunch and the beam frequency of 25 MHz, $f_{dia}$ = 625 ± 50 kHz. The intrinsic dark count of the SiPM, instead, was measured on the bench for the same threshold level of 6 p.e., and then corrected for the temperature difference between the laboratory and the MEDICYC experimental room; a value $DCR_{SiPM}$ = 302 ± 219 Hz was estimated. Thus, according to Eq. (2), $DCR_{coinc}$ = 3.8 ± 3.1 Hz. Despite the large experimental errors involved in the evaluation of $DCR_{coinc}$, its compatibility with $f_{noise}$ = 1.46 ± 0.02 Hz suggest that, the detection of particles other than PGs, if occurring, is certainly negligible in this time window.

A further proof of this observation is given by data presented in Fig. 8, left. In the experiment with 148 MeV protons, the PG module included a 6$\times$6 mm$^{2}$ SiPM that ensured a better optical photon collection efficiency with respect to the 3$\times$3 mm$^{2}$ SiPM used in the 63 MeV experiment. This allowed a higher threshold of 10 p.e. to be set, making the level of SiPM dark count rate negligible with respect to the data acquisition rate. The histogram in Fig. 8, left was built with a 15 ns coincidence window, but no data was acquired outside the 8 ns window defined by the bunch width. In summary, when the SiPM threshold is set high enough to cut-off the dark count contribution, the Cherenkov-based gamma detector is rather insensitive to neutron-associated background, and therefore the optimum candidate for the construction of a high sensitivity, fast PG detector.

Still, one last source of background remains in the collected data. Figure 10, showed two bumps in the TOF distributions, respectively in the ranges (−1.0 $\div$ 0.5) ns and (2.9 $\div$ 4.1) ns for detectors A and B. The origin of these events can be understood by TOF considerations: they cannot originate from in the target since the upstream detector (det A) measures for them a TOF smaller than the one measured for PGs originating from the target. Since these particles cannot be faster than PGs, they must come from upstream $\times$ the target. The TOF of these background events is rather well defined (suggesting a localized spatial origin) and it is compatible (for both the upstream and the downstream detectors) with protons directly travelling from the diamond detector to the PG module. Moreover, they appear in the same time-position of protons that are directly detected by the SiPM; the latter have a very particular shape and they can be easily identified (and rejected) by pulse-shape analysis. However, since protons of such energies cannot trigger Cherenkov production in the PbF$_2$, they must be converted locally into PGs in order to maintain their TOF coherence. In summary, our hypothesis is that these contributions are due to protons scattering in the diamond PCB board and interacting in the Cherenkov radiator holder made of Polyoxymethylene ($(CH_{2}O)_{n}$). The chemical composition of Polyoxymethylene is very close to that of PMMA, and results in the local production of PGs that are indistinguishable from the actual signal. Currently this effect cannot be rejected in the data, but it can be avoided in the future by improving the crystal holder design (or removing it altogether). We do not expect the PG conversion to take place in the crystal itself as we have not observed this effect in the 63 MeV experiment, for which the crystal was simply wrapped in a black tape, with no holders. Still, this hypothesis requires further experimental verification. In the light of this observation, it should also be observed that the scattered proton background must also affect data in Fig. 8.

## Discussion

Prompt gamma emission is a rare physical phenomenon, therefore, increasing detector sensitivity is of utmost importance to achieve treatment monitoring in real time with PG-based systems. Being able to measure the proton range *in vivo* with a limited PG statistics essentially means that the treatment can be verified very quickly at its very beginning and avoid unwanted over-irradiation of the healthy tissue.

Our work focuses on two main aspects: proposing a new PG imaging technique (PGTI) to improve the proton range measurement sensitivity, and conceiving an innovative PG imaging detector with high detection efficiency and high time resolution. The potential performances of PGTI have been discussed in a previous paper for different operating conditions. Here we focussed on the experimental feasibility of the PGTI technique in SPR in order to characterise the inherent performances of the proposed detection module. The SPR was realised in manual delivery mode since the clinical settings do not currently allow for such low intensities as they have no clinical applications yet. For the same reason, the dose delivery algorithm does not currently offer the possibility of switching from SPR to clinical intensity. While no technical barriers were identified in the delivery of the SPR intensity neither in terms of feasibility nor in terms of operation speed, it was observed that the SPR intensity is too low compared to the S2C2 ionisation chambers’ sensitivity. This would most probably require, in the future, the development of a dose monitoring system (e.g. a diamond beam monitor) dedicated to SPR as well as a new delivery software in addition to the current IBA “blind golfer” algorithm.

For 63 MeV protons we achieved, in SPR, a proton range sensitivity of 4 mm (at 2$\sigma$) with an unprecedentedly low statistics of only 600 PGs. This value confirms the 3 mm (at 2$\sigma$) predicted by MC simulation^18^ with 3000 PG events acquired. From these simulations, it can also be estimated that 600 PGs would correspond to 2 $\times$ 10$^{6}$ incident protons for the full 30-channels TIARA prototype (0.6% detection efficiency). This results paves the way to the use of the TIARA detector at the very beginning of the session to position the patient and/or verify the most critical spot(s) while operating a reduction of the beam intensity to $\sim$ 1 p/bunch. The duration of this monitoring procedure varies according to the time characteristic of the accelerator employed. For an accelerator such as the MEDICYC cyclotron, delivering 10$^{7}$ protons in SPR would require about 0.63 seconds according to theoretical calculations, whereas this time would be longer (31.6 s) for the S2C2 synchrocyclotron. The SPR approach should therefore be considered part of the patient set-up procedure (of the order of 15 minutes in the clinical practice) rather than as the treatment itself, for which PGTI should rather be implemented at nominal intensities (e.g. from $\sim$2000 to $\sim$2 million of p/bunch for the S2C2).

In the experiment with 148 MeV protons, we could compare the TOF distributions obtained with the detector in two different positions. We qualitatively showed that using multiple detector configurations is pivotal to obtain a uniform and increased detection efficiency (and eventually sensitivity) throughout the proton range. This requires the use of a dedicated reconstruction algorithm as PGTI in order to correct for the non-linearities introduced by the PG TOF term. This correction may not seem necessary when using conventional gamma detectors but, when using a detection system with 235 ps (FWHM) time resolution, it is essential in order to fully exploit its potential precision.

PGTI therefore goes hand in hand with the development of new detectors with optimised time resolution and detection efficiency. The most recent gamma detection module, composed of a 2 cm$^{3}$ PbF$_{2}$ and a 6$\times$6 mm$^{2}$ MPPC, has shown a time resolution below 167 ps (FWHM) for 148 MeV protons irradiations. With a larger photodetector surface compared to the previous module, it was possible to set a higher detection threshold thus ensuring that no dark count events were acquired. A high detection efficiency is guaranteed by the lack of a collimation system and by the optimisation of the SNR as Cherenkov radiators are rather insensitive to background particles (mainly neutrons). We are currently working on the detector packaging optimisation to avoid the detection of scattered protons and conceiving a mechanical system to hold multiple modules all around the patient. The latter should be able to cope with the patient table, by either building a sort of helmet for the patient, or placing some of the detectors behind the table. This second approach is possible as PGT/PGTI is not very sensitive to Compton scattering: scattered PGs are only very slightly delayed (few ps at worst) and they maintain their temporal coherence (c.f. Fig. 11).

This work was carried out under the hypothesis that every single proton could be tagged in time by selecting only 1-proton signals during the analysis. Experimentally, and with the current design of our beam monitor, this would require lowering the beam intensity to less than one proton per bunch^21^ in order to minimise the probability of 2- and 3-proton events; an approach that would further increase the duration of the monitoring procedure. In order to overcome this limitation, different solutions (software and hardware) are under investigation to tag in time each proton in the bunch. We are conceiving a dedicated algorithm that exploits the increased rise time and the different shapes of 2-, 3-, 4-proton signals to extract separate time stamps. The precision of these time stamps would be worse than those obtained for 1-proton signals (397 ps FWHM at S2C2), but they would still be more precise than the 2.7 ns FWHM CTR expected at nominal intensity for both PGT and PGTI. At the same time, we are developing a large area, multi-channel diamond-based beam monitor^21,33^ that would not only allow to overcome the size limitation of our current prototype, but also to tag in time multiple protons’ signals with the same precision of single protons^33,34^. The combination of a multi-channel monitor and a dedicated time tagging algorithm could result in a further extension of the beam intensity for the “single proton” regime. Still, once the protons’ time stamps are available a dedicated algorithm should be developed to iteratively (or through ML) identify the very proton that has produced the detected PG. As a result of this procedure, some events will have a degraded time-, and therefore space-resolution. A new assessment of the technique sensitivity will be therefore necessary in this scenario, but we expect to perform better, by design, than with nominal intensities by either PGT or PGTI.

Finally, it should be kept in mind that the SPR is a possibility, not a requirement for PGTI and for our detector. Our approach would allow to perform an *in-vivo* control of the patient set-up at the beginning of the treatment by verifying his/her anatomy, and then it could be used during the whole treatment at nominal intensities with performances that are not worse than PGT but with the advantage of employing Cherenkov detectors. At clinical intensity (e.g. from $\sim$2000 p/bunch for the S2C2), the loss in time resolution could be compensated by the increased acquisition statistics (see Jacquet et al.^18^ for details) to achieve similar sensitivities in the proton range measurement. At even higher intensities (the maximum intensity achievable with S2C2 is of $\sim$36$\times$10$^{6}$ p/bunch), Cherenkov radiators offer very good perspectives to sustain high count rates. The time-scale of Cherenkov process is of the order of the ps (to be compared to tenths of ns at best for conventional scintillators), resulting in a negligible dead-time, with the signal duration essentially given by the recharge time of the SiPM microcell. The latter can be cut-off to few ns with the appropriate electronics. In fact, the low-light output of the Cherenkov process ensures that only a few microcells per PG are activated, with the others available for the next event. It is therefore realistic to design a Cherenkov module that can sustain count rates up to $\sim$100 MHz per channel. At these extreme count rates, however, the design of an electronic board capable of tagging in time each PG is challenging and, at some point, different approches as the calculation of the center of gravity of the PG distribution^18^ should be used. For our final 30-channels prototype, we are conceiving a dedicated electronic, which will be based on digital TDCs, in order to handle the different regimes.

## Methods

### Single proton regime

For the 63 MeV experiment at the MEDICYC facility, the beam intensity was arbitrarily set to obtain a negligible ratio of 2-protons signals at the diamond level. The MEDICYC cyclotron is already calibrated to work down to a nominal intensity of 0.1 p/bunch.

At the S2C2 synchrocyclotron, the beam intensity depends on two parameters: the voltage of the Dees (V$_{Dee}$) and the S2C2 collimation slit opening. V$_{Dee}$ is given as a percentage of the maximum value. In the clinical practice the system calibration is performed for V$_{Dee}>$66.49%. In this work, the “effective” SPR at the beam monitor level required to set a V$_{Dee}$ of 65%. The slit opening, instead, was set to the minimal value of 1 mm. The spot integrity was verified in these conditions and no modifications were detected with respect to the clinical mode.

The SPR was performed in “manual delivery mode” for feasibility and safety reasons, so as to not corrupt the “clinical site configuration” of S2C2 which is extensively certified and validated for clinical purposes. This configuration, in fact, does not enable the SPR, as these intensities have no clinical application at this time and any modification of the settings would require the complete recalibration of the facility and a double validation (from both IBA and the customer).

### Effective beam intensity

The effective beam intensity at the beam monitor level was calculated *a posteriori* taking into account Poisson statistics. The diamond energy distribution was integrated in the regions corresponding to 0 and 1 proton signals to obtain the probability of having zero (P(0)) or one (P(1)) protons in the bunch. The ratio P(1)/P(0) provides the $\lambda$ parameter of the Poisson distribution describing proton delivery, which corresponds to the average number of protons per bunch.

Nevertheless, the intensity values calculated in this work do not correspond to the actual intensity set at the accelerator level. For the MEDICYC cyclotron, the calculation is biased by the presence of a collimator, whereas, for the S2C2 synchrocyclotron, the beam monitor covered only $\sim$20% of the beam surface, meaning that the actual beam intensity was of the order of 4.7 p/bunch .

### Intrinsic detection efficiency

A Geant4^35^ simulation (version 10.4.p02) of the optical properties of the TIARA module, based on the UNIFIED model and the QGSP-BIC-EMY physics list^17^, was performed to establish the module intrinsic detection efficiency as a function of the incident PG energy. The efficiency was computed as the fraction of PGs depositing more than 100 keV in the crystal and resulting in more than N$_{th}$ p.e. reaching the SiPM (with N$_{th}$ being the threshold expressed in p.e). The SiPM photodetection efficiency was also taken into account. Simulated data were then fitted with an analytical function (given by the sum of a sigmoid and a first degree polynomial) that was exploited to take into account the detector response in other MC simulations carried out for this work. The functions obtained for different values of N$_{th}$ are shown in Fig. 3.

### Generation of reference profiles

Reference profiles are built from MC simulations of the reference geometry. The Geant4.10.4.p02 version with the QGSP-BIC-EMY physics list was used to generate the PG time stamps on a detection surface of the same size as the gamma-detector module in order to take into account its geometrical detection efficiency.

The detector response was subsequently taken into account by considering the detection probability of each simulated event as defined by the analytical function presented in Fig. 3 for a threshold of 6 p.e (orange curve). The system TOF resolution was then included by convolving the data with a gaussian distribution of 315 ps FWHM (i.e. the experimentally measured value).

This multi-step approach was chosen to reduce the computing time, as PG generation is a rare phenomenon and the simulation of optical photon propagation and interactions in Geant4 are time-consuming. The excellent agreement between the experimental and simulated reference profiles in Fig. 5b and d is an indirect validation of the simulation procedure.

### Measurement of the distance between two PG profiles

After background rejection, the procedure used to measure the distance between two PG profiles is the same for PGT and PGTI (reconstructed) profiles. First, the reference X value $x_{ref}$ (either in the unit of time or space for PGT or PGTI respectively) is defined as the distal maximum in the simulated reference profile. Then, each experimental PG profile and the simulated PG profile are integrated to exploit the noise-filtering properties of the integral function. The difference $d_{i}$ between each experimental integrated PG profile ($f_{i}(x)$) and the integrated reference profile ($f_{ref}(x)$) is calculated as $d_{i}=f^{-1}(y_{ref})-x_{ref}$, where $y_{ref}=f_{ref}(x_{ref}$). This method is described in detail in Marcatili et al.^22^.

### Errors for the sensitivity plot

The error on the profile fall-off position (cf. Fig. 6) is determined from toy experiments, using the bootstrap technique. For each experimental TOF profile, 5000 sub-samples (toy experiments) including from 30 to 135 PGs (in steps of 15 PGs) were extracted, for a total of 8 sets of 5000 data samples per profile. The size of sub-samples was kept small to limit their statistical dependency. The 5000 sub-samples were then used to estimate the 1$\sigma$ and 2$\sigma$ statistical errors on the difference $d_{i}$ between the toy experiment profile and the reference profile, and to obtain their dependency on the number *N* of PG events in the profile. This dependencies varies as $k/\sqrt{N}$ (where *k* is a constant) and allows to extrapolate the experimental statistical errors at 1$\sigma$ and 2$\sigma$ for the PG statistics available in the current experiment.

### Background subtraction in PGTI distributions

The background in PGT distributions is flat as it is generated from events that are not time-correlated to the PG signal. When the PGT distributions are reconstructed, the flat background is non-linearly transformed acquiring a complex, non constant shape. A model of the PGTI background is built by reconstructing a constant signal according to Eq. (1). The model is then fitted on the reconstructed TOF histogram and subtracted the background.

It should be noted, that a more straightforward method would have been to subtract the flat background from the PGT profile before reconstruction. However, with the aim of implementing here an event-by-event reconstruction that could be performed as data are acquired, we performed the analysis under the assumption that the background level is not known at reconstruction.

## Data Availability

The datasets used and/or analysed during the current study are available from the corresponding author on reasonable request.
